# Observations on Fever

**Published:** 1828-05

**Authors:** 

**Affiliations:** Alnwick.


### FEVER.

*Observations on Fever.* Addressed to a Medical Friend, by Dr. Bow, of Alnwick.

(Continued from p. 300.)

In some work or other I have read as the opinion of its author, that the animal system was possessed of one dose of vitality, which could not become superabundant in one part without a proportionate deficiency in some other part, and vice versa. Such a law, I suspect, obtains with nervous influence. If there be too great a determination of it to one particular point or surface, it will be proportionally withheld from other parts; and if we diminish the sensibility of a part, so that usual impressions are not recognised, the usual force of influence is not transmitted to such part, and consequently it is transmitted to some other part or parts in greater force. If to the healthy subject we administer a brisk cathartic, we by it cause a determination of nervous influence greater than natural to the gut; therefore, during the operation of the medicine, there will be felt a greater or less degree of muscular debility and of coldness of the surface, feelings plainly arising from deficiency of nervous influence, owing to the demand for it elsewhere. Although the *general* action induced by a cathartic be sedative, the cathartic itself is a stimulant, and directly acts as such. On the other hand, if we administer a moderate dose of opium, we thereby diminish the sensibility of the stomach, and consequently the usual force of nervous influence is not elicited; it is, therefore, transmitted elsewhere in greater force, giving rise to hilarity of mind, increase in the force and frequency of the pulse, increased warmth of the surface, with moisture of the skin; yet, though these symptoms be demonstrative of excitation, they are not the immediate effects of the opiate. Opium is a direct sedative, acting upon the nervous extremities, producing a kind of torpor of them, by which they are more or less unfitted as instruments of transmission of impression to, or of influence from, the nervous centres. If the dose of opium be increased, there is torpor communicated to the brain itself, by which it becomes unconscious of all impressions, and sleep is the result. If opium act by diminishing the sensibility of the surface to which it is applied when administered internally, and if, in consequence of this effect, there be a greater determination of nervous influence to the surface of the body, we ought reasonably to expect that its external application would, by diminishing the sensibility of the skin, cause a greater determination of nervous influence to internal organs; and this we find to be the case. In debilitated habits, I invariably find that opium applied externally acts as a laxative, and I am not only in the habit of so applying it with the view of moving the bowels, but also of improving the appetite; effects very different, indeed, from those resulting from its internal administration.

If a patient labouring under typhous fever be left to himself, or if the treatment during the first stage, and three first days of the second stage, be inertly conducted, and if nature do not assist him by some critical discharge, then is he in danger of ulceration of the mucous and muscular coats of the intestines; a circumstance which happens so frequently, at least on the continent, as to have given rise to the celebrated though delusive doctrine of Broussais. I have said that nervous energy generally begins to decline on or after the third day of the second stage, after which time, if the irritation on the surface of the body continue, nervous influence will still continue to flow to the skin, though actually not in such great force as on the preceding days of the same stage, yet in greater force in proportion to that transmitted to other parts; for we find that, the weaker the patient, the more easily is the stream of nervous influence directed to or from any particular surface. Therefore, the irritation of the surface continuing, and as now nervous influence is more easily diverted, internal organs are in a greater degree deprived of it. And that the mucous membrane of the intestines should be the part to suffer in consequence of the diminished supply, is just what might have been expected from the distribution of the branches of the sympathetic nerves; for, although these nerves send branches to every part of the body, their principal expenditure is on these two extensive secreting surfaces, the skin and mucous membrane of the bowels: hence an agent acting on the one surface must necessarily induce a contrary action on the other. Indeed, either of these surfaces is so liable to be affected by causes operating on the other as to be a circumstance of daily observation. Nervous influence being then almost wholly diverted from the mucous surface of the intestines, infarction of its follicles, venous congestions, ulcerations, and gangrene follow. I have not been present at any dissection where the ulceration alluded to has been witnessed, nor have I seen a more accurate description of it than that by Dr. Hewett, in the London Medical and Physical Journal for August and September, 1826; and I think whoever peruses the cases and observations there given will at once recognise disorganization from want of action rather than ulceration consequent to inflammation. As a proof that this want of action arises from the determination of nervous influence to the surface, owing to the superior sensibility of the skin, I may observe that such appearances may be found to follow a preternatural determination of influence to the surface, however caused; for unless upon this principle, it would be difficult to explain the appearances found by Dr. Hewett in the young woman who died in consequence of extensive burn. If this view of the subject be correct, the conclusion to be drawn from it is of the utmost importance in practice. With the view of turning the stream of nervous influence inwardly, I frequently employ opium externally during the second stage of fever, and from that I ever get what I expected, viz. an improved state of the stomach and in the appearance of the tongue; and as I must declare my astonishment at the rapid recoveries of some, may I not believe that, by determining nervous influence to the intestines, I have unwittingly accomplished a more essential point, that of arresting or preventing the ulcerative disorganization of the mucous coat of the intestine?

The perusal of Mr. Ward's communications to the first and some of the succeeding volumes of the London Medical and Physical Journal led me to the employment of opium externally in fevers, and I know not why the cases and facts stated by him have not urged the generality of practitioners to adopt the practice; unless, indeed, the supposed *modus operandi* of opium. For if opium acted only on the system after having gained the circulation, the effects following its exhibition would be the same in every instance. But, as this is not the case, there being a very sensible difference in the effects of its internal and external application, and as the mere mode of its introduction, whether by lymphatics, lacteals, or otherwise, is inadequate to explain the difference, perhaps this difference may be supposed, by those who favor the idea of absorption, to rest more on fancy than on truth.

In prolonged fevers, the low muttering delirium, the restlessness, and the denied sleep, are, I think, caused by the deficient secretion of the fluid destined to lubricate the meninges of the brain. If such be the case, we can readily discern why we may succeed in procuring quiet sleep by opium externally applied, when, internally administered, it might aggravate the symptoms we mean to remove. For, by applying opium to the skin, we diminish its sensibility, we produce a torpor of the extremities of the sentient nerves, by which a preternatural determination of nervous influence to the surface is prevented. This nervous influence being now determined inwards to parts much in need of it, the lubricating secretions become more copious, irritation is diminished, and sleep follows.

But it is not the secretions only which become more copious or more perfect in consequence of the morbid determination of nervous influence to the skin being put a stop to; for the patient awakes with an appetite for the food which before he loathed, proving that the gastric fluid hath become more copious or more perfect. The mahogany colour of his cheek is changed to a brighter red, proving that the change of the blood in the lungs hath become more perfect. His pulse, which was running towards an hundred and twenty, weak and irregular, is now regular and below an hundred, proving that the determination of nervous influence to the heart is now sufficient to regulate its action; although this difference in the action of the heart may, in some measure, proceed from the more perfect arterialization of the blood.

To understand why opium might aggravate the symptoms we intended it to remove, when given internally, I have only to refer you to what I have said of its *modus operandi* in the healthy subject. It determines nervous influence to the surface of the body. Now, the symptoms we wish to remove are owing, in a great measure, to a morbid determination of nervous influence to the surface. If by the greatness of the dose we succeed in procuring sleep, it is far from being quiet or sound, and the patient awakes unrefreshed: nay, it is proverbial that, in such cases, benefit seldom arises from sleep effected by opium. Insensibly have I been led to make the above and many other remarks not immediately hinging on my subject, yet, as I conceive they are not altogether out of place, I trust you may also be of that opinion.

I will now propose to you the following condensed hypothesis, which will at one glimpse give you my idea of the chain of actions which constitutes fever:

*Impaired energy and functional disorder of the nervous system, and, in consequence, prostration of strength and imperfect secretion generally; irritation from the imperfect products of secretion, and, in consequence, increased nervous energy and sense of heat; absorption of the imperfect products, and, in consequence, inordinate vascular action.*

The first link of the above, as you must remark, is also the first link of the Cullenian theory. I have employed the expression "nervous system" instead of brain, and perhaps such alteration was unnecessary; but of that more hereafter. I have added "functional disorder," because, by doing so, I think I meet one objection which has been started, viz. that diminished energy of the brain is not necessarily followed by fever. I can readily suppose that the energy of the brain may be impaired to a degree even greater than that necessary to produce fever, without fever being the result; but this impairment must be so gradual as at first to effect the secretions but triflingly, and then, instead of fever, we have, according to circumstances, a disease to be found in the class Cachexia. I regret I can go no further with Cullen; for I think it is needless to call in the aid of an ideal power in the animal economy to perform indirectly that which we can conceive to be performed directly by a simple and natural process; a process which we often artificially imitate to effect the same end.

One of the functions of the nervous system being secretion, or rather (to make use of language which may not offend an anti-neurologist,) the brain having an influence over the secretory apparatus, I have noted as a consequence of impaired energy imperfect secretion generally; and, as I have already said, it is owing to the imperfect state of the secretions throughout the body that a hot stage follows the cold one. The irritation produced by these imperfect products is the cause of the reaction, and in the greater sensibility of the skin we have the reason why the reaction is first observable on the surface. If imperfect secretion generally be admitted as a consequence of impaired nervous energy, it follows that the imperfect products must not only irritate, as would foreign matter, the nervous extremities with which they come in contact, but also that they must be absorbed and enter the circulation likewise in the shape of foreign matter. And if these imperfect products be sufficient to irritate, and to produce inconvenience, as soon as they are separated from the blood, in the parts with which they come in contact, it is but reasonable to conclude that, when absorbed, they will excite, by irritation, the heart and arteries to increased action.

It is impossible for me to foresee all the objections you may start to the above hypothesis, for, as the father is frequently the only one who is blind not only to the moral, but even to the physical, defects of his child, so I, aware of my partiality, must look to you to point to its failings: it fails not, however, in simplicity, and it speaks neither of unmeaning sympathies, of morbid associations, nor of imagined *vires medicatrices naturæ.*

As I am one of those who believe that all fevers are produced by the same proximate cause, I meant here to show the adaptation of the above theory (if I may so name it) to some particular forms of fever; but, with a view of bringing this communication to an end, and upon the consideration that, if it explain the phenomena of the common continued fever of this country, it will be equally explanatory of the leading characters of all fevers, I will reserve what I have to say for some future epistle. Yet there is one form of fever which I cannot avoid adverting to in this place, inasmuch as it stands recorded to this day as an insuperable objection to the Cullenian theory; to the very stage which alone is correct, and which I have borrowed as the basis of my own. It is said that in symptomatic fever there are no symptoms of impaired energy of the brain. Be it so; and partly owing to that it is that I have said "nervous system" in my condensed view, instead of "brain," as written by Cullen. No acute inflammation can possibly happen in any part of the body, unless there be a preternatural determination of nervous influence to the part; consequently, there must be a deficiency of it in other parts of the body, and, owing to this deficiency, the secretions of such parts are imperfectly produced. These imperfect products at length irritate, are absorbed, and fever established. Thus, though this fever does not commence with symptoms of depression so marked as to constitute signs of impaired energy of the brain; yet I presume it will not be denied that there is disorder of the nervous system, consisting in impaired energy generally, the increased local determination being its remote cause. We have now a fever corresponding in its leading features to the stage of excitement in idiopathic fever. This fever is not of long continuance, for the absorbed offending matter, after a time, is determined to, and finds an outlet at, the wound or injured part, in the form of pus. The symptomatic fever I allude to is that which alone should be called so, viz. that which surgeons recognise as such.

The fevers said to be produced by the local affections called phlegmasiæ is, for the most part, if not always, an idiopathic fever. It is an inflammatory fever in which, owing to certain causes, an irregular distribution of nervous influence gives rise to topical inflammation. In the same way topical inflammation obtains in typhus, but here we never think of calling the fever symptomatic.

